# Doctors Have no Right to Refuse Medical Assistance in Dying, Abortion or Contraception

**DOI:** 10.1111/bioe.12288

**Published:** 2016-09-22

**Authors:** Julian Savulescu, Udo Schuklenk

**Keywords:** autonomy, professionalism, interests, justice, law, euthanasia

## Abstract

In an article in this journal, Christopher Cowley argues that we have ‘misunderstood the special nature of medicine, and have misunderstood the motivations of the conscientious objectors’.^1^ We have not. It is Cowley who has misunderstood the role of personal values in the profession of medicine. We argue that there should be better protections for patients from doctors' personal values and there should be more severe restrictions on the right to conscientious objection, particularly in relation to assisted dying. We argue that eligible patients could be guaranteed access to medical services that are subject to conscientious objections by: (1) removing a right to conscientious objection; (2) selecting candidates into relevant medical specialities or general practice who do not have objections; (3) demonopolizing the provision of these services away from the medical profession.

## *Reasonable Arguments*’ Lack of Traction Explained

Cowley begins his defence of conscientious objection by conceding: ‘Schuklenk's arguments appear very reasonable, and his concern for patients is genuine. What he and Savulescu lack is an explanation of why their arguments have failed to move legislators or professional bodies.’^2^

To the extent that this is true, the answer is obvious: the influence of organized religion in society. The more religious a society is, the more religious values are imposed on people. Many of the conscientious objection protections we are grappling with today were written into constitutional arrangements in times gone by when the influence of churches was significantly more powerful than it is today. In strongly Christian societies, like Ireland, abortion remains illegal. Indeed, in Ireland, symphysiotomy was still performed into the 1980s in preference to Caesarean section for obstructed pregnancy. This involved barbaric splitting of the pubic symphysis to allow the passage of the head of the baby. This resulted in horrific side effects for the woman, including gross incontinence, pain and restricted mobility, but was performed in many cases without consent, without even informing the patient, in part because Catholic doctors believed that a Caesarean section might impede the woman's ability to have the maximum number of children possible in the future.^3^

In fact, Cowley is wrong that no countries have rejected a right to conscientious objection. Enlightened, progressive secular countries like Sweden, have labour laws in line with our arguments. Sweden provides no legal right of employees to conscientious objection.^4^ Employees could be sacked for failing to provide legal services under labour law. The same holds true, for instance, for Finland, where reportedly ‘CO to participating in induced abortion is not present in the Finnish health care system or legislation unlike in many other European countries.’^5^ This solves the problem of patient access to care, and it has not had a detrimental effect on applications to these countries’ medical schools, or to the ability of their medical professions to replenish their ranks with new graduates. These countries have resolutely prioritised patient access to care over the protection of doctors’ idiosyncratic moral convictions with regard to these services. Other countries ought to follow Sweden and Finland.

## ‘Weak Arguments’ and Weak Arguments

Cowley then moves on to give what he admits are ‘weak arguments’.^6^ The first is that where the objectionable practice is a very small part of a GP's work, we should accommodate a refusal to perform it, as we should accommodate a GP with a back complaint who requires a change in duties for medical reasons. He gives a number of reasons to reject this argument but misses the central one: the provision of these services is a good thing.

Let us consider this argument in the case of contraception, another standard focus of treatment deniers (conscience objectors). If contraception accounts for a small amount of a GP's practice, should we accommodate a conscientious objection to it?

Conscientious refusal to provide contraception is common and mistakenly supported by medical boards and medical associations (see for instance recent Australian reports).^7^

But contraception is legal because the ability to control reproduction is one of the greatest and most valuable of human achievements. Before modern contraception, women died early, suffered from multiparity, were chained to the home, could not work or get an education. When we make contraception legal, we do not do so merely because people *ought* to be free to choose when and how many children to have. It is because it is *good* to choose this.

Contraception is a social good (at least in a world with a sufficient population), and it is demanded by eligible autonomous patients, and insofar as doctors have a monopoly over its provision, they ought to provide it.

For religious GPs, obstetricians and pharmacists to refuse to provide the oral contraceptive pill is simply unprofessional. There is no requirement for a healthcare system to accommodate unprofessional behaviour: there is no analogy with accommodating individual occupational health needs.

One problem in countries like Australia, Canada, the UK and the United States face is that they have historically made provision for conscientious objection. Therefore people who enter medicine with a religious belief against standard medical practices such as contraception do so with an expectation that they will be able to conscientiously object. It might be argued then that this should be honoured.

It is unclear, however, why this initial expectation should be decisive in practical policy making. A career in medicine might span 40 years and the field a doctor leaves might be almost unrecognizable from the field she enters. It is clear that the scope of professional practice is ultimately determined by society, and that it is bound to evolve over time. That is true not only for the question of what kinds of services must be provided, it is also true for conscientious objection itself, as the mentioned examples of the two Scandinavian countries show. If a professional norm is no longer fit for purpose, it should be changed.

Regardless of how one views the matter of grandfathering‐in those who signed up to practising medicine believing that they would have the indefinite right to refuse, for example, to provide contraceptive services, we should create a system that guarantees patients access to contraception because it is good. Whether it is a small part of a given doctor's job is irrelevant. If it is part and parcel of a doctor's professional role to provide contraception, they should provide it.

Therefore, even if we did not change the system for those already practising medicine, given that there is an oversupply of people capable and willing to become medical professionals, we should select those willing to provide the full scope of professional services, and those who are most capable. Medical schools and training programmes should carefully outline the nature of the job and screen for conscientious objection where it is relevant to job performance. Requirements of the job should be written into the contract.

This is, in effect, what Sweden has done. There might be risks in requiring people to provide services they don't want to provide: perhaps some or even many of them might do so half‐heartedly, possibly to the detriment of patient care. However, there are no data from countries such as Sweden and Finland to suggest that that this concern has any basis in reality. Doctors who did behave like this would be acting with a gross lack of professionalism and would therefore be subject to censure and appropriate remedies by their professional, statutory bodies. At any rate, the best way to address such a concern is better ethics education. In addition to selecting candidates who are tolerant, have epistemic responsibility and humility and are willing to accommodate patient values,^8^ we need medical ethics education to encourage better understanding of values and their place in medicine.

Finally, if conscientious objection continues to be tolerated in medicine and results in treatment denial, alternative ways of guaranteeing reasonable and fair access to these goods ought to be provided. One way of doing this is to de‐monopolize the provision of the relevant service. If the quality of such a service could match that provided by medical professionals, and sufficient supply could be ensured, then this would be a viable alternative. This would require new training, selection, regulatory and oversight procedures, which would be cumbersome and expensive. But there is no reason why only doctors could competently provide, for example, contraception, abortion or assisted dying services.

## The Importance of Moral Integrity

Cowley proceeds to advance Wicclair's moral integrity argument, albeit in a qualified way:

According to the first prong of the argument, being forced to act against one's conscience can result in a loss of integrity, and this in turn leads to ‘strong feelings of guilt, remorse, and shame as well as a loss of self‐respect’ (26), to people dropping out of the profession or, if they know their integrity will not be protected, to not entering the profession in the fist [*sic*] place.’^9^

If selection processes make it clear that conscience claims cannot be deployed and as a result people who object on grounds of conscience to certain aspects of the job are not selected, or choose not to pursue a career in medicine, would this make for worse medical practice?

We don't know of any evidence that those with religious beliefs make better medical doctors. If it were the case that Christians or Muslims, or members other religious groups, who are conscientious objectors, make better doctors because of these ideological mindsets, this would be a reason to accommodate conscience in selection procedures. We are deeply sceptical that holding religious beliefs makes one better at the practice of medicine. Of course, there are many characteristics that a good doctor displays in addition to purely technical or scientific knowledge and skills. This might include traits such as empathy and compassion. However, these are traits that are not specific to religious people, nor do all religious people possess them.

On the contrary, as the example of symphysiotomy shows, some religious beliefs, when imposed on medical practice, can have a highly detrimental effect. Other examples include the resistance of the Catholic Church to providing birth control in developing countries as a part of aid, and its opposition to the provision of condoms to prevent the spread of HIV in sub‐Saharan Africa and prisons or the suboptimal care provided by religiously motivated healthcare professionals to gay and lesbian patients.^10^ Richard Dawkins got it right, when he noted, ‘religion is not simply vicars giving tea parties. There are evil consequences.’^11^ We should evaluate medical practices not on their basis in religion, but on their impact on the patient.

One might object that this response misses the point. One might grant that it would be better for patients in the future if doctors did not conscientiously object, or if they were admitted to the profession in the first place. But nonetheless, we face a problem now that there are doctors who do conscientiously object and respecting their integrity gives them the prerogative not be involved in its provision.^12^

Doctors must put patients' interests ahead of their own integrity. They must ensure that legal, beneficial, desired services are provided, if not by them, then by others. If this leads to feelings of guilty remorse or them dropping out of the profession, so be it. As professionals, doctors have to take responsibility for their feelings. There is an oversupply of people wishing to be doctors. The place to debate issues of contraception, abortion and euthanasia is at the societal level, not the bedside, once these procedures are legal and a part of medical practice.^13^

## Analogies, Directness and Moral Responsibility

Supporters of conscientious objection in Western literature typically confine their analysis to a discussion of Christian conscience. They often struggle with other religions, particularly Islam, whose conscientious objectors include those who will not treat the opposite sex at all, and can barely conceive of secular consciences that might influence medical treatment.

Savulescu gave the example of a doctor who subscribes to the fair innings argument that people over the age of 80 have had their fair share and should not receive life prolonging medical treatment. This is a defensible value: Daniel Callahan, Ezekiel Emanuel,^14^ amongst others, have supported it. Nonetheless, these personal convictions should not govern bedside care or intensive care practice in the absence of some kind of general democratic societal endorsement.

Cowley sees this is as a telling analogy so he tries to show why it is disanalogous. He gives two reasons.

The two cases differ in the directness of the contemplated wrong. In the abortion context, the objecting doctor is very clear about the nature of the moral wrong that she is being asked to authorise or perform, and she is very clear about the victim of the wrongful act. On the other hand, it is less clear that such a ‘good‐innings’ doctor, in being forced to override her objection and treat an over‐80 patient, thereby commits or even colludes with what she considers wrong; the wrong, for her, is the more abstract one of some other, younger patient whose treatment has perhaps been postponed because of the proposed use of expensive scarce resources on this over‐80 patient. And it would be a stretch to say that this doctor is responsible for that younger patient's suffering or death, when there are so many other causally relevant institutional factors between them.^15^

However, ‘directness’ is morally irrelevant.^16^ The belief that it is morally relevant is a psychological bias that causes enormous damage in everyday life. The fact that we do not know the identity of the victims of our acts or omissions does not change their gravity. We can easily imagine a real life case where there is a shortage of intensive care beds. If an 85 year old were admitted now, the unit would be full. Based on experience we know with near certainty that there would be a younger patient, perhaps a car accident victim, needing another bed tomorrow. The fact that we do not know the identity of this patient at the time of decision‐making does not alleviate responsibility for that person dying if no bed can be found. The following example serves to better illustrate this point:

Imagine you are an executioner. You shoot a condemned person to death. You caused the death. Now imagine you are a part of 10 man firing squad. You all manage to hit your target – the heart – at the same instant. You are not 10% morally responsible for the death now. You should put down your rifle if you believe the person is innocent, or the death penalty unjust, even if you know 9 others will kill the person.^17^

The multiplicity of causal factors does not alleviate moral responsibility. It is a feature of ordinary moral psychology to think that we are less responsible if many people contribute to a catastrophic outcome. For this same psychological reason, people don't feel responsible for climate change – their contribution is negligible even though together the effects could be enormous.

In any case, if society thinks contraception, abortion and assistance in dying are important, it should select people prepared to do them, not people whose values preclude them from participating. Equally, people not prepared to participate in such expected courses of action should not join professions tasked by society with the provision of such services.

Cowley continues his argument,

In addition to the problem of determining causality here, there are two very different perspectives in play: the good‐innings argument is ultimately focused on changing the resource distribution policy; if there were enough resources, that same doctor might well treat the over‐80 patient. On the other hand, the anti‐abortion doctor adopts a local policy when she contemplates the destruction of the innocent human being in the woman in front of her, regardless of the impact that such a conscientious objection might have on the rest of policy.’^18^

We believe the analogy stands. To show this, we will now offer two cases of secular conscientious objection where doctors are complicit in a direct lethal wrong involving identifiable patients.

## Two Cases of Secular Conscientious Objection

Here are two unethical practices that doctors are forced to be complicit with and where the law ought to change.

The first is the destruction of human embryos created in the course of assisted reproduction when these could be used by another infertile couple or for research to develop cures for life‐threatening diseases. We believe that patients should not be able to choose to destroy their embryos when their family is complete but rather should give them either to other infertile patients^19^ or to medical research or treatment. This is a deeply held value. Yet the law in the UK and Australia requires their destruction after a certain number of years. We consider this law to be unethical.

How should IVF specialists respond who share our views? Here we arrive at the role of conscience and the place of values in medicine. The law is such that couples can or must destroy their excess embryos. We believe these laws should change. This should motivate us to campaign to change them. In the meantime, doctors should engage patients in argument and try to convince them rationally, using evidence, to donate their excess embryos.^20^ But in the end, if couples wish to destroy their excess embryos, IVF specialists should comply, regardless of their conscientious objection to the practice.

Another example of immoral behaviour is the refusal to donate one's organs after death. We have argued that there is a minimal moral duty to donate organs.^21^ It is a zero‐cost rescue and can save up to 10 lives.

As consequentialists, we view failing to donate organs as tantamount to killing innocent people. We believe it is deeply wrong to not donate organs after death and rather choose to bury them or burn them.

We have campaigned to change the law to an opt‐out regulatory regime, permitting society to override family refusal and to prioritise organ donors in the allocation of scarce transplant organs.^22^ In the absence of a change of existing regulatory regimes doctors should try to convince patients to donate their organs, but if in the end, they refuse, that refusal should be respected even though we do not believe they have a morally defensible claim to those life‐preserving organs after death. Doctors thus should be complicit with lethal immorality.

It is a common objection to our view that doctors are not instruments but moral agents. It is correct that doctors are moral agents like any one of us who is capable of making moral choices. The place of reasons and values in medicine is properly located in dialogue with patients,^23^ and in attempting to shape policy and law, as we have done in the case of changing the law around abortion,^24^ and euthanasia.^25^ However, we are not entitled to impose those values on patients in the delivery of health care and deny treatment when these patients are legally entitled to access that particular service.

It is Cowley, then, who has misconceived the nature of medicine and the role of values in medicine in a democratic society by suggesting that doctors’ personal moral conviction should take priority over other considerations, such as patients’ legal rights or the importance of a rational dialogue with patients.

## Moral Wrongness in Medicine

Cowley is confused about the role of moral judgment in the delivery of medical services. In differentiating objection to providing abortion from objection arising from racist or homophobic discrimination, he claims views about the moral status of the fetus are legitimate because they are in some sense defensible. He writes:

In contrast, a doctor can refer to the wrongness of abortion as an intelligible reason for refusing a patient, without thereby losing moral and intellectual respect. There is a real debate to be had about abortion, whereas there is no debate about racism. Abortion is one of several legitimate ‘fault lines’ in the public moral consensus, where each side has a prima facie respectable point of view, and there are no grounds for thinking that one side is necessarily ignorant or prejudiced in some way.^26^

Cowley is also confused about the role of judgments of moral wrongness in medicine and the delivery of medicine. One can (and some or many do) view the following things as morally wrong:

1. Contraception
2. Abortion
3. Euthanasia
4. Failing to donate your organs
5. Smoking, drinking and taking drugs
6. Over or under eating
7. Engaging in premarital or homosexual sex or sex with multiple partners
8. Destroying excess embryos that could be used by other couples or researchers
9. Keeping people with profound dementia alive by artificial feeding
10. Having a hospital birth or an elective Caesarean section or a homebirth
11. Gender assignment or reassignment surgery for intersex conditions or gender dysphoria
12. Providing HPV vaccine to adolescent girls

The list could go on and on. As one of us has argued elsewhere, individual moral judgments about the rights and wrongs of particular medical practices are by necessity partly arbitrary. They are arbitrary in the sense that their moral basis cannot be conclusively evaluated for soundness (an impossibility when it comes to religious convictions, for instance). In some of these cases, there can be reasonable disagreement about whether the practice is right or wrong. As a result of this, pretty much any conscience view that is the result of some deliberation and is claimed to be held deeply and sincerely ‘counts’. This view is, in fact, shared by the Courts in most English speaking jurisdictions.^27^ Cowley and others like him have no means to distinguish the conscientious objection to providing medical aid in dying from providing IVF to homosexual patients (where the objection is to perceived complicity in same‐ sex parenthood rather than to IVF per se). It is true that societies draw lines in the sand with regard to what kinds of views they accommodate at a certain point in history, but these lines are by necessity at least partly arbitrary, too. The only means Cowley could use to explain why medical aid in dying and abortion are ‘different’ would be an argument not from conscience but from tradition. Arguments from tradition carry, of course, no normative weight.

If a service a doctor is requested to perform is a medical practice, is legal, consistent with distributive justice, requested by the patient or their appointed surrogate, and is plausibly in their interests, the doctor must ensure the patient has access to it. It is then irrelevant how defensible the doctor's own moral take on the patient's actions is. We might even agree with the doctor over whether a particular practice is morally wrong. For all practical intent and purposes that is irrelevant to their obligations as professionals *vis‐a‐vis* the patient.

## Ethical Relativism and Evil

At the heart of the paradox of conscientious objection is arguably a belief in ethical relativism.^28^ Ethical relativism is probably the dominant view of ethical statements in the lay and professional public. Put simply, value is in the eye of the beholder. Ethical relativism is the view that the truth of ethical statements ‐ such as abortion is wrong ‐ is dependent on, or relative to, the culture, group or individual making the statement. Ethics is relative to groups, cultures or times.

But ethical relativism is practically ethical nihilism. If one accepted ethical relativism, the holocaust was, from the Nazi's perspective, right. It is just that today we have a different set of values from the Nazis. As the Nazi example demonstrates, we have reason to be profoundly uncomfortable with ethical relativism, even though it is often considered the politically correct thing to be an ethical relativist.

Part of the force behind respecting conscientious objection is a common commitment to ethical relativism: if that is what someone believes, then they are right to believe it, and that alone makes it a kind of truth.

But we cannot escape arguments over what is right when we discuss conscientious objection. We do want doctors to act on their conscience when the stakes are high, and their conscience is right: when they are asked to be complicit in or participate in an evil. Nor do we want what might loosely be called ‘unconscientious objection’. That is, doctors mistakenly failing to provide beneficial care on the basis of false moral beliefs. This is the paradox of conscientious objection.

Here is one case of a valid conscientious objection that deserves our respect. A United States Navy nurse refused to force feed Guantanamo detainees, including Dhiab, an inmate who was detained in Guantanamo from 2002 until December 2014, despite being approved for release by the US government in 2009. Dhiab reported the nurse's actions as part of a legal case against the force‐feeding. According to Dhiab's account the nurse initially allowed prisoners not to be force fed if they were ill, but later refused to participate at all. The nurse subsequently underwent a number of internal investigations, including the threat of court martial, before being returned to duty earlier this year.^29^

Why do we applaud this conscientious objector, but reject others, who may hold as strong views about abortion as this nurse did about torture? It is trivially true that torture is not a medical practice which is in the interests of the patient or desired by the patient. Nor is it legal. The argument in support of this conscientious objector then is that the navy nurse was asked to perform a procedure that was not within the scope of nursing practice. The patients in question were competent and refused the procedure in question. The combination of the procedure not being within the scope of professional practice, and the patient not wanting the procedure shows why this case does not map onto the conscientious objection cases that we are concerned about. To reiterate: What justified the nurse's refusal to participate in the act of torture was first and foremost that force‐feeding was (and is) not part and parcel of nursing's professional scope of practice. It would have been unprofessional to force‐feed the objecting prisoner. The objection was justified on professional grounds.

What if the contested practices really *are* evil? That is, if we reject ethical relativism, but we are objectively wrong about the morality of one or more of these cases.

We have used contraception as the major example throughout the article. It is an area where conscientious objection is applied. However, for many people it is not a matter of any moral significance. For that reason, Cowley and others primarily use abortion as their example: it is an issue over which there is significantly greater discomfort. So what if we are right about contraception and the case of conscientious objection overall, but in fact we are wrong about abortion, and the practice of abortion is indeed evil, and akin to torture?

In that case, our current conscientious objection policies are in any case untenable: they only succeed if we agree to ethical relativism. If authorizing an abortion (for example) were really in itself evil (both objectively and significantly immoral), it is hard to see how proposed referral policies could be tenable. If the practice is evil, the individual should not be any part of it, even by being a member of that speciality or profession. If a doctor views abortion as an evil, she should not be a gynaecologist or GP.

There is a long debate about the morality of abortion, which we do not have space to cover here. But most people who believe contraception, abortion and euthanasia are wrong don't believe they are evil in the same way as, for example torture or genocide are evil. If its rightness or wrongness is of a type or degree that it is a matter of personal preference (ethical relativism), it should not have an impact on patient care. If it is objectively evil then the solution conscientious objection offers is inappropriate.

Cowley's solution, while common in policies and regulations in liberal Western democracies, is often sold to the public as a reasonable compromise between those denying conscientious objection rights and those who insist on a right to refuse the provision of such medical services. Cowley writes: ‘the GP can still refer to one of her colleagues in the knowledge that she (the GP) is not responsible for her colleague's free actions, she is merely describing a fact – a widely available fact, and hardly a secret – of what her colleague is willing and able to do.’^30^ This strategy is clearly an unjustifiable compromise from the perspective of the objector. If you believe that abortion constitutes the murder of a human person, a ‘compromise’ that would oblige you to pass the pregnant women on to a colleague who you know would be willing to commit the ‘murder’, evidently does not constitute a viable compromise. If abortion were not just something that an individual happens to disagree with but is objectively evil, then she should do everything she can to stop her patient having an abortion.

There is another way in which relativism is relevant to debates about conscientious objection. It is relativism about the purposes of medicine. Cowley expresses this when he writes eloquently, but mistakenly, in relation to the ‘calling’ of medicine:

In other words, it is not a contingent aversion to abortion that she happens to hold, it is not a psychological quirk that can and should be overcome, it is not some debating‐society posture that should be abandoned in the ‘real world’, and it need not even be theologically motivated. Rather, it has to do directly with the nature of medicine as she understands and identifies with it, an understanding that makes abortion objectively incompatible with being a doctor as healer. For her, pregnancy is not simply a disease or injury that needs medical treatment. The important thing to stress here is that this understanding of medicine is not at all bizarre or idiosyncratic.^31^

He goes on to praise the moral consciousness of conscientious objectors: ‘they deserve accommodation not out of respect for their integrity, but rather out of respect for their conception of medicine.’^32^

Breast‐beating stuff to be sure, but substitute ‘contraception’ for ‘abortion’ and this is indeed a ‘bizarre and idiosyncratic’ view of medicine. For example, ‘[aversion to contraception] has to do directly with the nature of medicine as she understands and identifies with it, an understanding that makes [contraception] objectively incompatible with being a doctor as healer.’

If you don't believe contraception or sterilisation are part of the modern practice of medicine, don't become a GP. As one of us has argued elsewhere, even if there were a strong ‘calling’ to medicine or to a particular field within medicine, people are still free to decline the call and do something else with their lives. If they were not free to make that choice, due to the strength of the ‘call’, it is questionable that their decision to join the medical profession was truly an autonomous choice in the first place.^33^ It is arguable that people with this view of their relationship to medicine would not be ideally suited to join the medical profession. They have a higher power that they are serving first in their medical practice, their vocation, which has taken away their freedom to make informed choices. That makes a mockery of their graduation promise to serve the patient interest first and foremost: their understanding of their vocation will always take priority.

## Secular Conscientious Objection Alive and Well and how Secular Doctors Ought to Reign in Their Values Too

Today there is a shortage of organs for transplantation purposes. Despite recent advances in assistive technologies that have halved the waiting list mortality rate for heart transplants, around 8% of children still die on the waiting list.^34^ Children with chromosomal abnormalities such as Down Syndrome and Trisomy 18 sometimes require heart transplantation following heart failure related to congenital abnormalities. Because these children have intellectual disability, in some cases severe, they are sometimes not placed on the waiting list for heart transplantation. Reasons for not doing so can include medical reasons such as the ability to understand and follow post‐operative treatment regimes,^35^ although increasingly, good outcomes from transplantation are reported.^36^ However, a number of cases have been reported where the refusal appears to be an expression of a particular view of distributive justice held by their treating doctors that discounts the quality or the value of life of those with intellectual disabilities against those without when calculating the distribution of resources to achieve the greatest good.^37^

However, this practice is in direct contravention of the stated principle of most international guidance and principles of egalitarianism that underpin public health systems. Such children need a heart transplant and should have an equal chance of getting one.^38^

Here we have a secular conflict between some doctors' values (utilitarianism) and a stated health care policy (egalitarianism). Following our logic, such doctors ought to place children with Down's Syndrome and Trisomy 18 on waiting lists for heart transplantation and give them an equal chance of such a transplant. If they believe principles of distributive justice are being applied unjustly in current healthcare policy, they should campaign for such a reform in their governing policy. At present, a doctor who refused to apply policies which consider medical need only in allocating resources would be unjustly discriminating against their patient. Failure to list for transplantation children with intellectual disability, even in severe cases such as Trisomy 18, based on utilitarian values is a secular example of objectionable conscientious objection.

## Lessons from the Netherlands?

Cowley lauds the success of the Dutch system of a register of doctors willing to perform euthanasia.^39^ As he notes, this works because there are plenty of doctors, knowledge of such a register is widespread and the Netherlands is a small country. But is this an appropriate solution to the problems caused by conscientious objectors? To create a register of doctors willing to prescribe contraception? Why should scarce health care resources be expended on such a system only to support what constitutes unprofessional conduct by health care professionals? Why should patients seeking a simple script for a prescription contraceptive be inconvenienced in their choice of doctor by such unprofessional conduct?

The problem with conscientious objection is that it has been freely accommodated, if not encouraged, for far too long.

It is important to understand that when doctors have a monopoly over a procedure like surgery, it is not a luxury that they can choose to give or withhold on personal grounds. There are criteria around justice, autonomy and interests that determine whether it is provided. When contraception, abortion or euthanasia are made legal and they become part and parcel of medical services over which doctors have monopoly power, patients do acquire a right to them.

## Pluralism About Interests?

There is a last resort for the conscientious objector as there is for any doctor who seeks to enforce his or her values *vis‐a‐vis* the patient. This is called paternalism. This is to claim the intervention is not in the patient's interests. Cowley clutches at this last straw:

There is another way to put this. I referred above to the patient's right to medical treatment under a public health system. But this does not include a right to a particular treatment, e.g. one whose miraculous properties the patient has just read about on the Internet. The patient has a right to medical attention to her symptoms and problems, but it will be for the doctor, using her expertise, skills and judgement, to decide on the most appropriate course(s) of treatment. And it will be up to the hospital or health service management to decide whether such a treatment represents good value for public money. This is a well‐established principle of medical discretion. The same principle can be applied to the case of PAS in a jurisdiction such as the Netherlands where it is legally permissible. The patient presents herself to the doctor and demonstrates her fulfilment of the six conditions, and informs her of her wish to commit suicide. Under the principle of medical discretion, it is for the doctor to decidewhether PAS is or is not the most appropriate ‘treatment'.^40^

It is bizarre to liken medical aid in dying to a treatment on the internet purported to have miraculous effects. It is entirely clear what euthanasia is and what it will achieve. And it is likely to be very good value for public money when the alternative is continued medical or social care given against a competent patient's considered wishes.

But what of the claim that it is not in the patient's best interests? In the case of euthanasia, this is among the more powerful arguments against providing it.

There are, however, three possible responses to this objection to the provision of euthanasia. The first is to argue interests should be subjectively defined. If a person says their life is not worth living, it is not worth living. This argument is problematic^41^ – people can be mistaken about what is good for them and indeed whether their life really is worth living. Precisely our valuing autonomy might justifiably lead to the temporary overriding of occurrent autonomy in order to preserve dispositional autonomy, eg in cases of anorexia or morbid obesity.^42^ However, if the wish remains stable over time, and the patient's view of their quality of life remains stable, then such a strong paternalistic approach becomes incompatible with the values predominant in liberal democracies.

The second is to give up the idea that medicine is only about promoting objective best interests. It is importantly also about respecting patient autonomy. It is then sufficient to show that a procedure is justified by distributive justice and the person autonomously desires it. For example, sterilisation of a young professional woman may not be in her interests but should still be provided if she autonomously desires it.

Thirdly, patients in liberal Western democracies clearly have a right to suicide, including by refusal of eating and drinking.^43^ Given that they will be able to end their lives if they pursue this course of action, their interests are better served by medical aid in dying. This is because medical aid in dying would result in a better death for such a patient than him‐ or herself starving to death or dying of dehydration.^44^

This discussion is, ultimately, irrelevant in jurisdictions that have decriminalized assisted dying. Legislators or courts, always with overwhelming public support, have determined that particular patients have a right to access medical aid in dying. If patients meet the standards defined in the relevant legislation, they are entitled to access medical aid in dying, regardless of what a particular doctor thinks about their ‘best interest’.

## Conclusion

Reasons and values are essential to medicine. Doctors like others should have values that reasonably track what is right. Individual values ought not to govern delivery of health care at the bedside. Doctors can campaign for policy or legal reform. They can also provide advice with reasons, based on their values. But they have no claim to special moral status that would permit them to deny patients medical care that these patients are entitled to.

## Footnotes

2
Ibid: 359.

3
O. Walsh. Report on Symphysiotomy in Ireland 1944–1984. *Department of Health, Ireland* 2014 Available at: http://health.gov.ie/wp-content/uploads/2014/07/Final-Final-walsh-Report-on-Symphysiotomy1.pdf [Accessed 7 June 2016].

4
U. Schuklenk & R. Smalling. Why medical professionals have no moral claim to conscientious objection accommodation in liberal democracies. *J Med Ethics* 2016; doi:10.1136/medethics-2016-103560; F. Cranmer. Sweden, abortion and conscientious objection: Ellinor Grimmark. *Law & Religion UK* 2015. Available at: http://www.lawandreligionuk.com/2015/11/17/sweden-abortion-and-conscientious-objection-ellinor-grimmark/ [Accessed 7 June 2016]. 10.1136/medethics-2016-10356027106748

5

P. Nieminen , S. Lappalainen , P. Ristimäki , et al. Opinions on conscientious objection to induced abortion among Finnish medical and nursing students and professionals. BMC Med Ethics 2015; 16:17 doi: 10.1186/s12910-015-0012-1. 2588569810.1186/s12910-015-0012-1PMC4376492

6
Cowley. *op. cit*. note 1. p. 359.

7
C. Zielinski. 2015. Australian doctors can deny women the contraceptive pill. Is this OK? *News.Com.Au* 13 March. Available at: http://www.news.com.au/lifestyle/health/australian-doctors-can-deny-women-the-contraceptive-pill-is-this-ok/news-story/ace48c27497173f356d4a5aaaf54c772 [Accessed 7 June 2016].

8
For further elaboration on the virtues of moral doctors, see J. Savulescu . Liberal Rationalism and Medical Decision‐Making. Bioethics 1997; 11: 115–129. 1165479110.1111/1467-8519.00049

9
Cowley, *op.cit*. note 1, p. 360.

10
U. Schuklenk. 2009. Human Self‐Determination, Biomedical Progress, and God. In. *50 Voices of Disbelief – Why We Are Atheists* R Blackford and U Schuklenk, eds. Wiley: Blackwell: Chichester, 323–331. U. Schuklenk, R. Smalling. Queer patients and the health care professional – Regulatory arrangements matter. *J Med Humanit* 2013; 34(2): 93–99. U Schuklenk. Public health ethics and the law of the land. *Developing World Bioethics* 2011; 11(1): ii‐iii. 10.1007/s10912-013-9211-223443248

11
N. Cohen. 1994 Profile: Darwin's disciple: Who needs God when we have biology? Nick Cohen meets the scourge of theologians. *The Independent*, 01 January. Available at: http://www.independent.co.uk/voices/profile-darwins-disciple-who-needs-god-when-weve-got-biology-nick-cohen-meets-the-scourge-of-1397467.html [Accessed 30 May 2016].

12
We thank Robert Card for this objection.

13

J. Savulescu . Conscientious Objection in Medicine. BMJ 2006; 332: 294–297 doi: 10.1136/bmj.332.7536.294; 1645573310.1136/bmj.332.7536.294PMC1360408

14
E. J. Emanuel. 2014. Why I hope to die at 75. *The Atlantic*, October. Available at: http://www.theatlantic.com/magazine/archive/2014/10/why-i-hope-to-die-at-75/379329/ [Accessed 30 May 2016]; D. Callahan. 1998. *Setting Limits: Medical Goals in an Aging Society*. Simon and Schuster.

15
Cowley. *op. cit*. note 1, p. 361.

16

P. Singer . Famine, Affluence, and Morality. Philos Public Aff 1972; 1: 229–243.

17
Of course, we do not believe conscientious objectors are like executioners in one important way: they do not kill a person. This analogy illustrates the nature of collective responsibility. If a conscience objector did believe she was killing an innocent person, this analogy shows she should not only put down her gun, but do everything she could to prevent the execution, as we will go on to argue.

18
Cowley. *op. cit*. note 1, p. 361.

19

G. Fuscaldo and J. Savulescu . Spare Embryos: 3000 Reasons to rethink the significance of genetic relatedness. Reprod Biomed Online. 2005; 10 (2): 164–68. Available at: www.rbmonline.com/Article/1550 Online: 21 December 2004. [Accessed 30 May 2016]. 1582321710.1016/s1472-6483(10)60936-4

20
Savulescu, *op. cit*. note 8.

21

W. Isdale & J. Savulescu . Three proposals to Increase Australia's Organ Supply. Monash Bioeth Rev 2015; 33(2):91–101. 2616953010.1007/s40592-015-0030-2

22

U. Schuklenk . Why I should be allowed to sell a kidney. Kingston Whig‐Standard 2013, July 19. Available at: http://www.thewhig.com/2013/07/19/why-i-should-be-allowed-to-sell-a-kidney [Accessed 30 May 2016]; Isdale and Savulescu. *op. cit*. note 21.

23
Savulescu. *op. cit* note 8; J. Savulescu. Rational Non‐Interventional Paternalism: Why Doctors Ought to Make Judgements of What Is Best for Their Patients. *J Med Ethics* 1995; 21: 327–31. 10.1136/jme.21.6.327PMC13768288778455

24
L. De Crespigny & J. Savulescu, J. Pregnant Women with Fetal Abnormalities: The Forgotten People in the Abortion Debate. *Med J Aust* 2008; 188(2):100 – 102; L. de Crespigny& J. Savulescu. Abortion: Time to Clarify Australia's Confusing Laws. *Med J Aust* 2004; 181(4):201–203.

25

U. Schuklenk , J.J. van Delden , J. Downie , et al. End‐of‐life decision‐making in Canada: The report by the Royal Society of Canada Expert Panel on end‐of‐life decision‐making. Bioethics 2011; 25 (suppl 1): 1–73. 10.1111/j.1467-8519.2011.01939.xPMC326552122085416

26
Cowley. *op. cit*. note 1, pp. 360–361.

27
Schuklenk & Smalling, *op. cit*. note 4.

28

A. Giubilini . The paradox of conscientious objection and the anemic concept of conscience. Kennedy Inst Ethics J 2014; 24 (2): 159–185. 2510909410.1353/ken.2014.0011

29
C. Rosenberg. 2016. Navy reinstates nurse who refused to force‐feed at Guantánamo. *Miami Herald* 3 May. Available at: http://www.miamiherald.com/news/nation-world/world/americas/guantanamo/article75398072.html [Accessed 30 May 2016]; C. Rosenberg. 2014. Navy nurse refuses to force‐feed Guantánamo captive. *Miami Herald* 15 July. Available at: http://www.miamiherald.com/news/nation-world/world/americas/article1975643.html [Accessed 30 May 2016].

30
Cowley. *op. cit.,* note 1, p. 362.

31
Ibid: 362.

32
Ibid.

33
Schuklenk & Smalling, *op.cit*. note 4.

34

F. Zafar , C. Castleberry , M.A. Khan et al. Pediatric heart transplant waiting list mortality in the era of ventricular assist devices. J Heart Lung Transplant 2015; 34: 82–8. 2544757410.1016/j.healun.2014.09.018

35
Down's Heart Group. Heart/Lung Transplants. *Down's Heart Group…. Supporting a Better Life* Available at: http://www.dhg.org.uk/information/heartlungtransplants.aspx [Accessed 7 June 2016].

36

C.A. Irving , M.P. Chaudhari . Cardiovascular abnormalities in Down's syndrome: spectrum, management and survival over 22 years. Archives of Disease in Childhood 2012; 97: 326–330. 2183583410.1136/adc.2010.210534

37

T. P. Shriver . The discriminatory reason doctors won't give a baby the heart she needs. The Washington Post 2016, April 8. Available at: http://www.syracuse.com/opinion/index.ssf/2016/04/infant_wrongly_being_denied_heart_transplant_because_of_possible_developmental_d.html [Accessed 7 June 2016]; Ibid.

38

A. Wightman , J. Smith , D. S. Diekema . Neurodevelopmental Status as a Criterion for Solid Organ Transplant Eligibility. Ethical Issues in Pediatric Organ Transplantation. Springer 2016 pp 215–236.

39
Cowley. *op. cit*., note 1, pp. 362–363.

40
Ibid: 363.

41

J. Savulescu . Concise argument – wellbeing, collective responsibility and ethical capitalism. J Med Ethics 2016; 42:331–333. 2722252110.1136/medethics-2016-103674

42

R. Young . 1986 Personal autonomy: Beyond negative and positive liberty. London: Croom‐Helm.

43
B. White, L. Willmott, & J. Savulescu. Voluntary Palliated Starvation: A Lawful and Ethical Way to Die? *J Law Med* 2014; 22: 276–286; J. Savulescu. A Simple Solution to the Puzzles of End of Life? Voluntary Palliated Starvation. *J Med Ethics* 2014; 40:110–113.

44
J. Savulescu. 2015. Autonomy Interests, Justice and Active Medical Euthanasia. In *New Directions in the Ethics of Assisted Suicide and Euthanasia* M. Chobi & J. Varelius. Eds. International Academy of Law, Ethics, and the New Medicine Series, Cham: Springer, pp.14–58; J. Rachels. Active and Passive Euthanasia. *New Engl J Med* 1975; 292:78–80. 10.1056/NEJM1975010929202061109443

